# Effects of *Brugmansia arborea* Extract and Its Secondary Metabolites on Morphine Tolerance and Dependence in Mice

**DOI:** 10.1155/2012/741925

**Published:** 2012-02-08

**Authors:** Laura Mattioli, Antonio Bracci, Federica Titomanlio, Marina Perfumi, Vincenzo De Feo

**Affiliations:** ^1^Pharmacognosy Unit, School of Pharmacy, University of Camerino, Via Madonna delle Carceri 9, 62032 Camerino, Italy; ^2^Department of Pharmaceutical and Biomedical Science, University of Salerno, Via Ponte don Melillo, 84084 Fisciano, Italy

## Abstract

The aim of the present study was to investigate, *in vivo*, the effect of a *Brugmansia arborea* extract (BRU), chromatographic fractions (FA and FNA), and isolated alkaloids on the expression and the acquisition of morphine tolerance and dependence. Substances were acutely (for expression) or repeatedly (for acquisition) administered in mice treated with morphine twice daily for 5 or 6 days, in order to make them tolerant or dependent. Morphine tolerance was assessed using the tail-flick test at 1st and 5th days. Morphine dependence was evaluated through the manifestation of withdrawal symptoms induced by naloxone injection at 6th day. Results showed that BRU significantly reduced the expression of morphine tolerance, while it was ineffective to modulate its acquisition. Chromatographic fractions and pure alkaloids failed to reduce morphine tolerance. Conversely BRU, FA, and pure alkaloids administrations significantly attenuated both development and expression of morphine dependence. These data suggest that *Brugmansia arborea* Lagerh might have human therapeutic potential for treatment of opioid addiction.

## 1. Introduction

*Brugmansia arborea *(L.) Lagerh. is a solanaceous shrub native to South America and widely cultivated in Europe as an ornamental species. In Peru this plant, known with the vernacular names of campachu or misha, is employed by the shamans in magic and sorcery *to get in touch with the gods*, as an antinflammatory and in the treatment of rheumatic pains. Misha is one of the most powerful magical plants, a “hot” species, known to act on the central nervous system [1].

Previous phytochemical studies identified some active components of the plant. The tropane alkaloid, hyoscine, was found in samples collected in Argentina [2]. Other tropane alkaloids have been found in plants collected in Italy [3].

Few pharmacological studies are available in the literature about this plant. Extracts, chromatographic fractions, and pure alkaloids showed inhibitory activity on contraction of isolated guinea pig ileum induced both electrically and by acetylcholine, showing a spasmolytic activity *in vitro* [3]. *B. arborea* extracts, chromatographic fractions, and pure isolated compounds have been reported for their activity on CNS [4]. *In vitro* studies demonstrated the affinity of methanol and water extracts of the plant for 5-HT1A 5-HT2A, 5-HT2C, D1, D2, *α*1, and *α*2 receptors in binding assays [5, 6]. These biological systems are widely involved in the phenomenon of dependence. Particularly opioid addiction is certainly among the most widely diffused with a high rate of mortality.

Therefore, considering that *B. arborea *extracts and pure alkaloids are able to reduce the morphine withdrawal *in vitro* [7], the aim of the present study was to investigate the possible activity of a methanol extract, its chromatographic fractions, and three pure isolated compounds from *B. arborea* on the expression and the acquisition of morphine tolerance and dependence in mice.

## 2. Materials and Methods

### 2.1. Plant Material

Aerial parts of *B. arborea *were collected in June 2008 in Conca dei Marini (Salerno, Italy). The plant was identified by Dr. V. De Feo. A voucher specimen of the plant (labeled as DF/246) was kept at the Herbarium of the Faculty of Pharmacy, University of Salerno.

### 2.2. Extraction, Separation, and Identification

One kilogram of leaves and flowers of *B. arborea *were oven-dried at 40°C and powdered. The powder was extracted at room temperature with methanol for two days. The extract was concentrated *in vacuo*, giving 32 g of residue. This crude extract was called BRU. An aliquot of 3.4 g of this extract was purified on a Sephadex LH 20 column, eluting with MeOH. One hundred fifteen fractions were obtained and combined into 15 major fractions, on the basis of their chemical similarity as revealed by thin-layer chromatography (TLC). Fractions 3 and 4, containing alkaloids, were purified by RF-HPLC. Fraction 3 was purified using a C_18_ *μ*-Bondapak column, with the following conditions: flow rate 2.0 mL/min, eluent MeOH : H_2_O, 7 : 3; from this fraction pure apoatropine (4.7 mg) was obtained. From fraction 4, purified by RF-HPL, using a C_18_ *μ*-Bondapak column, with the following conditions: flow rate 2.0 mL/min, eluent MeOH : H_2_O, 6 : 4, atropine (19 mg) and 3*α*-tigloyl-oxitropane (12.3 mg) were obtained. Pure compounds were identified by accurate NMR analyses and comparison of their spectral data with data available in literature [8, 9]. The crude extract (BRU), the fraction containing alkaloids (FA), the fraction not containing alkaloids (FNA), and the pure compound (atropine, apoatropine, 3*α*-tigloyl-oxitropane) were texted on mice to study the possible interaction whit morphine.

### 2.3. Animals

Male CD-1 mice (Harlan SRC, Milan, Italy) weighing 25 g to 35 g were used. These mice were kept in a dedicated room, with a 12 : 12 h light/dark cycle (lights on at 09:00), a temperature of 20°C to 22°C, and a humidity of 45% to 55%. They were provided with free access to tap water and food pellets (4RF18, Mucedola, Settimo Milanese, Italy). Each mouse was used in only one experimental session. Ethical guidelines for the investigation of experimental pain in conscious animals were followed, and procedures were carried out according to EEC ethical regulations for animal research (EEC Council 86/609; D. Lgs. 27/01/1992, No. 116), and the testing was carried out according to the European Community Council Directive for the Care and Use of Laboratory Animals of 24 November 1986 (86/609/EEC).

### 2.4. Drugs

BRU is the denomination of crude methanol extract not purified. This extract (BRU: 7.5, 15, and 30 mg/kg/5 mL), its alkaloidal and nonalkaloidal fractions (FA and FNA at 5.5 and 24,5 mg/kg, resp.) and single pure compounds (atropine, apoatropine, and 3*α*-tigloyl-oxitropane, at 2.2, 1.8, and 1.5 mg/kg, resp.) were dissolved in water immediately before use and injected intraperitoneally (i.p.). The same vehicle was administered to control group. Morphine hydrochloride (Salars S.p.A, Como, Italy) was dissolved in saline immediately before use and was administered subcutaneously (s.c.) at a dose of 10 mg/kg. Naloxone hydrochloride (Sigma, St. Louis, MO), dissolved in water immediately before use, was administered i.p. at dose of 5 mg/kg.

### 2.5. Induction of Morphine Tolerance

According to Abdel-Zaher et al. [10] and Mattioli and Perfumi [11], morphine tolerance was induced in mice treated with the administration of morphine (10 mg/kg; s.c.) twice daily at 12 h intervals for 5 days. Tolerance was evaluated by testing the antinociceptive response to morphine on tail flick test on the 5th day, 30 min after the last morphine injection, in comparison with the 1th day. Briefly, tail flick test consists of the irradiation of the lower third of the tail with an I. R. source (Ugo Basile, Comerio, Italy). The basal predrug latency, ranged between 2-3 s, was calculated as the mean of two trials performed at 30 min interval. Then mice received tested-compound or related vehicle, 30 min before morphine or saline administration. The antinociceptive activity was evaluated, on the 5th day, 30 min after the last morphine injection. A cut-off latency of 12 s was established to minimize tissue damage. Antinociceptive effect was expressed as a percent of the Maximum Possible Effect (MPE%) according to the following formula:

(1) $$\begin{array}{c} \text{\%MPE}=\frac{\left(\text{Post-drug}\;\text{latency-baseline}\;\text{latency}\right)}{\left(\text{cut-off}\;\text{value-baseline}\;\text{latency}\right)}\times 100, \end{array}$$

where postdrug latency is tail-flick latency 30 min after the last morphine dose.

### 2.6. Effects of BRU on the Expression and the Acquisition of Analgesic Tolerance to Morphine

In order to evaluate the effect of BRU on the acquisition (development) of morphine tolerance, the mice (*n* = 8–12 for each group) were i.p. administered with BRU (7.5, 15 and 30 mg/kg), twice daily, 30 min before each morphine treatment. Its effects on the expression phases of morphine tolerance were evaluated in morphine-treated mice receiving acute administration of BRU, 30 min prior to the last morphine injection on test day (day 5). The effects of BRU on the development and the expression of morphine tolerance were evaluated by testing the analgesic effect of morphine on tail-flick test as described in detail. In addition, the antinociceptive effect of BRU alone on antinociception was examined, by tail-flick test, in nondependent mice that received single or repeated i.p. administration of different doses of BRU (7.5, 15, and 30 mg/kg).

### 2.7. Effects of FA and FNA on the Expression and the Acquisition of Analgesic Tolerance to Morphine

In order to evaluate which fraction of the total extract was responsible of the effect of BRU, the effects of both FA and FNA were tested on the expression and acquisition of analgesic tolerance to morphine.

Therefore mice (*n* = 8–10 for each group) received acute or repeated administration of FA (5.5 mg/kg), FNA (24.5 mg/kg), or related vehicle following the experimental procedure reported above. The doses of chromatographic fractions were chosen on their percentage on 30 mg/kg of BRU.

### 2.8. Effects of Single Alkaloids on the Expression and the Acquisition of Analgesic Tolerance to Morphine

The three alkaloids, isolated from the alkaloid-containing fraction (FA), were also tested on the expression and acquisition of analgesic tolerance to morphine. For this purpose, mice (*n* = 10 for each group) were administered with atropine, apoatropine, 3*α*-tigloyl-oxitropane at doses of 2.2, 1.8, and 1.5 mg/kg, respectively, as reported above. The dose of each alkaloid was chosen on their percentage on 5.5 mg/kg of FA.

### 2.9. Induction of Morphine Dependence

To develop dependence, the mice were treated with morphine (10 mg/kg; s.c.) twice daily at 12 h intervals for 6 days [10, 12]. Two hours after the last dose of morphine, withdrawal syndrome (abstinence) as an index of morphine dependence was precipitated by intraperitoneal injection of naloxone (5 mg/kg) [10, 12]. The combination of morphine with a high dose of naloxone on day 6 has been demonstrated to induce more severe symptoms, including autonomic signs, since naloxone precipitates dose-dependent withdrawal symptoms in animals acutely or chronically dependent upon morphine [12]. Ten minutes before naloxone treatment, the mice were placed in a transparent acrylic cylinder (20 cm diameter, 35 cm high) to habituate them to the new environment. Immediately after naloxone challenge, each mouse was again placed gently in the cylinder and then monitored for 15 min for the occurrence of withdrawal signs (jumping, rearing, forepaw tremor, and teeth chatter). The withdrawal symptoms are reported as a summary of all of the signs that were seen.

### 2.10. Effects of BRU on the Expression and the Acquisition of Morphine Dependence

To examine the effects of total extract on morphine dependence, BRU (7.5, 15, and 30 mg/kg) was given in mice (*n* = 8–12 for each group) i.p. chronically 30 min prior to each morphine injection (acquisition) or acutely before naloxone (expression) as described above. In addition, the effect of BRU alone on naloxone-induced jumping behaviour was examined in nondependent mice. Animals received single or repeated administration of different doses of BRU (7.5, 15, and 30 mg/kg; i.p.) or vehicle (5 mL/kg; i.p.). The assessment of naloxone-precipitated jumping behaviour after administration of BRU was already described in detail.

### 2.11. Effects of FA and FNA on the Expression and the Acquisition of Morphine Dependence

To examine the effects of the two fractions of the total extract on the expression and the acquisition of morphine dependence, FA (5.5 mg/kg) and FNA (24.5 mg/kg) were tested on naloxone-precipitated withdrawal syndrome behaviour as reported above (*n* = 8–10 animals for each group).

### 2.12. Effects of Single Alkaloids on the Expression and Acquisition of Morphine Dependence

Finally single isolated alkaloids (atropine, apoatropine, and 3*α*-tigloyl-oxitropane) were administered at the dose of 2.2, 1.8, and 1.5 mg/kg, respectively, based on their percentage on the alkaloidal fraction FA, in order to evaluate their effect on the expression and development of morphine dependence (*n* = 10 for each group). Experimental procedure was the same reported above.

### 2.13. Statistical Analysis

The statistical analysis was performed using two-way split-plot analysis of variance (ANOVA), with treatment as the between-subject factor, and time as the within-subject factor, to analyze morphine tolerance. The morphine dependence data were analyzed by one-way analysis of variance (ANOVA). When appropriate, *post hoc* analysis was carried out using a Newman-Keuls test, to determine the differences between groups. Statistical significance was set at *P* < 0.05, and the data are expressed as means ± S.E.M.

## 3. Results

The bioassay-oriented study of a methanol extract of *Brugmansia arborea* permitted the isolation of three tropane alkaloids: atropine, apoatropine, and 3*α*-tigloil-oxitropane. This result agrees with the available literature that reports that the genus *Brugmansia* contains this class of alkaloids [3].

### 3.1. Effects of BRU on the Expression and the Acquisition of Analgesic Tolerance to Morphine

**Figure 1:**
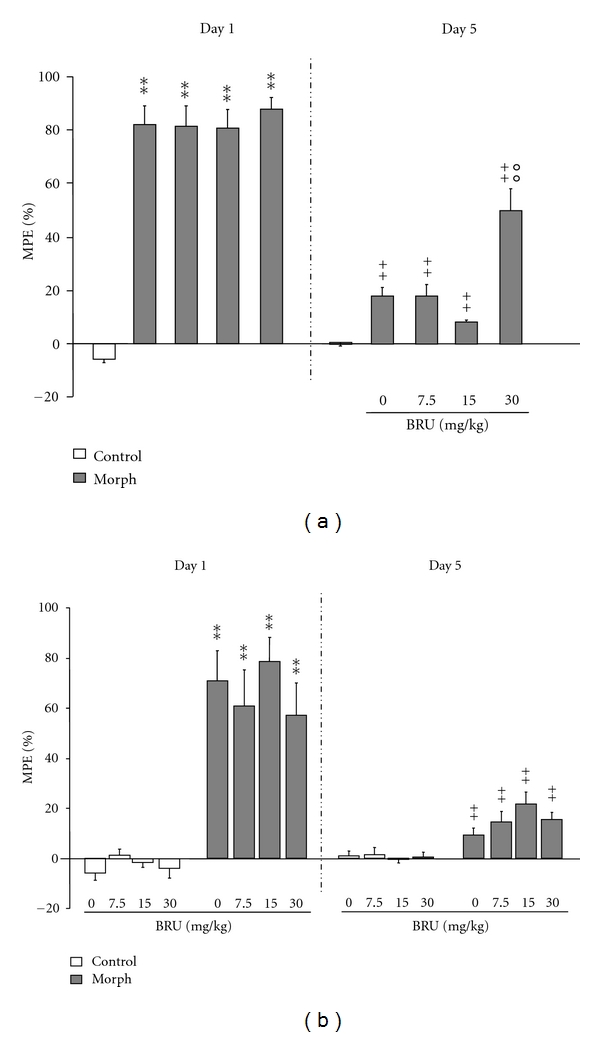
*Effects of different doses of Brugmansia arborea L. on the expression *(a)* and the acquisition *(b) *of tolerance to morphine-induced analgesia.* Mice were treated twice daily for 5 days with either saline or 10 mg/kg morphine. BRU (0, 7.5, 15 and 30 mg/kg) was administered 30 min before each morphine injection (acquisition) or prior to the last morphine treatment (expression). Morphine antinociceptive effect (%MPE) was assessed on day 1 and day 5, as indicated. Significant differences: **P < 0.01, compared to control group; ^++^ *P* < 0.01, compared to related-morphine group on day 1; °°*P* < 0.01, compared to morphine group on day 5.

The effects of BRU on the expression and acquisition of tolerance to morphine-induced analgesia are shown in Figure 1 (Panel (a) and (b), resp.). About expression data, overall analysis of variance revealed a statistically significant treatment and time effect [*F*(4,45) = 45.091, *P* < 0.001; *F*(1,4) = 204.415, *P* < 0.001, resp.], and interaction time × treatment effects were seen on analgesia latency in the tail-flick test [*F*(4,45) = 19.716, *P* < 0.001]. The mice treated with morphine showed a maximal antinociceptive effect (%MPE) on day 1 (*P* < 0.01 between control and morphine groups). Repeated s.c. administration of 10 mg/kg morphine twice daily to morphine-treated mice induced a significant decrease in the analgesia latency, on day 5 in comparison to that on day 1 [*F*(1,18) = 154.333; *P* < 0.001] (Figure 1(a)). Acute administration of BRU (7.5, 15, and 30 mg/kg) 30 min before morphine injection on the test day produced a significant decrease in the expression of morphine tolerance as compared to morphine-vehicle group. Particularly, the *post hoc* analysis revealed a statistically significant effect at the highest dose of 30 mg/kg (*P* < 0.001) (Figure 1(a)).

Acute administration of BRU to nondependent mice (control) did not modify the analgesia latency of the treated mice (*P* > 0.05) (data not showed). Concerning the effect of BRU on the acquisition of analgesic tolerance to morphine, overall analysis of variance revealed a statistically significant treatment and time effect [*F*(7,74) = 22.523, *P* < 0.001; *F*(1,7) = 52.156, *P* < 0.01, resp.], and interaction time × treatment effects were seen on analgesia latency [*F*(7,74) = 10.253, *P* < 0.001] (Figure 1(b)). The mice treated with morphine showed a maximal antinociceptive effect (%MPE) on day 1 [*F*(7,74) = 17.783; *P* < 0.001]. *Post hoc* analysis revealed that acute coadministration of BRU with morphine did not modulate the analgesia at any of the doses tested on day 1 compared to vehicle-morphine group (*P* > 0.05). Repeated s.c. administration of 10 mg/kg morphine alone twice daily to the mice induced, on day 5, a significant decrease in the analgesia latency in the tail-flick test, which resulted in a 70% reduction [*F*(1,18) = 41.204; *P* < 0.001] (Figure 1(b)). Pretreatment of the mice with 7.5, 15, and 30 mg/kg BRU 30 min before each morphine injection did not inhibit the development of tolerance to morphine analgesia at any of the doses tested (*P* > 0.05) (Figure 1(b)). Finally, repeated administration of BRU to nondependent mice (control) did not modify the analgesia latency of the treated mice (*P* > 0.05) (Figure 1(b)).

### 3.2. Effects of FA and FNA on the Expression and the Acquisition of Analgesic Tolerance to Morphine

**Figure 2:**
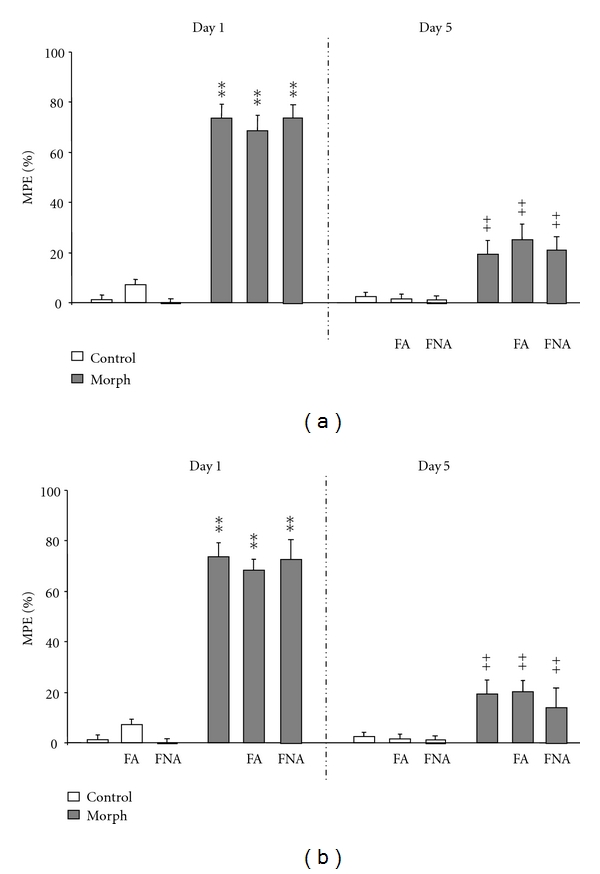
*Effects of FA and FNA on the expression *(a)* and the acquisition *(b)* of tolerance to morphine-induced analgesia.* Mice were treated twice daily for 5 days with either saline or 10 mg/kg morphine. FA (5.5 mg/kg) and FNA (24.5 mg/kg) were administered 30 min before each morphine injection (acquisition) or prior to the last morphine treatment (expression). Morphine antinociceptive effect (%MPE) was assessed on day 1 and day 5, as indicated. Significant differences: **P < 0.01, compared to control group; ^++^ *P* < 0.01, compared to related-morphine group on day 1.

As shown in Figure 2, both single (Panel (a)) and repeated (Panel (b)) administration of FA (5.5 mg/kg) and FNA (24.5 mg/kg), 30 min before morphine injection on the test day, did not produce significant prevention or treatment of the morphine tolerance (*P* > 0.05). In fact FA and FNA did not affect morphine-induced antinociception elicited by single or repeated dose injection, as confirmed by statistical analysis (*P* > 0.05).

### 3.3. Effects of Single Alkaloids on the Expression and the Acquisition of Analgesic Tolerance to Morphine

**Figure 3:**
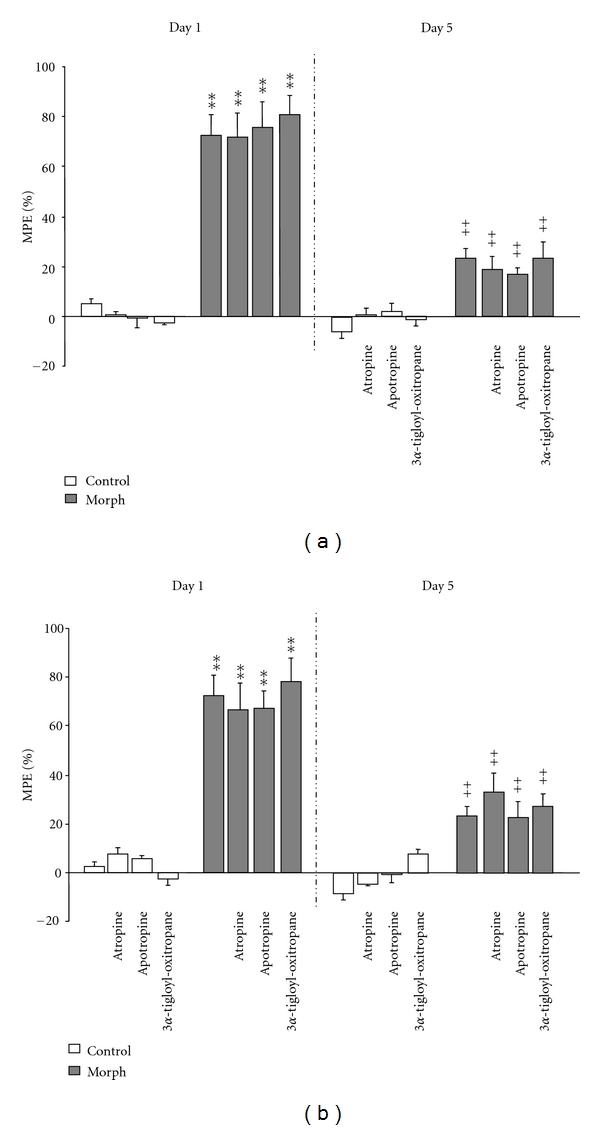
*Effects of single alkaloids on the expression *(a)* and the acquisition *(b)* of tolerance to morphine-induced analgesia. *Mice were treated twice daily for 5 days with either saline or 10 mg/kg morphine. Atropine (2.2 mg/kg), apoatropine (1.8 mg/kg) and 3*α*-tigloyl-oxitropane (1.5 mg/kg) were administered 30 min before each morphine injection (acquisition) or prior to the last morphine treatment (expression). Morphine antinociceptive effect (%MPE) was assessed on day 1 and day 5, as indicated. Significant differences: **P < 0.01, compared to control group; ^++^ *P* < 0.01, compared to related-morphine group on day 1.

Figure 3 shows the effects of atropine, apoatropine, and 3*α*-tigloyl-oxitropane on the expression (panel (a)) and the acquisition (Panel (b)) of analgesic tolerance to morphine. Two-way ANOVA followed by post hoc analysis revealed that there is no significant difference in MPE% of morphine among alkaloids-treated groups and vehicle-treated mice both in the expression [*F*(7,64) = 45.963, *P* < 0.001] and the acquisition [*F*(7,64) = 36.520, *P* < 0.001] test. Finally, repeated administration of atropine, apoatropine, and 3*α*-tigloyl-oxitropane to nondependent mice (control) did not modify the analgesia latency of the treated mice (*P* > 0.05) (Figure 3(b)).

### 3.4. Effects of BRU on the Expression and the Acquisition of Morphine Dependence

**Figure 4:**
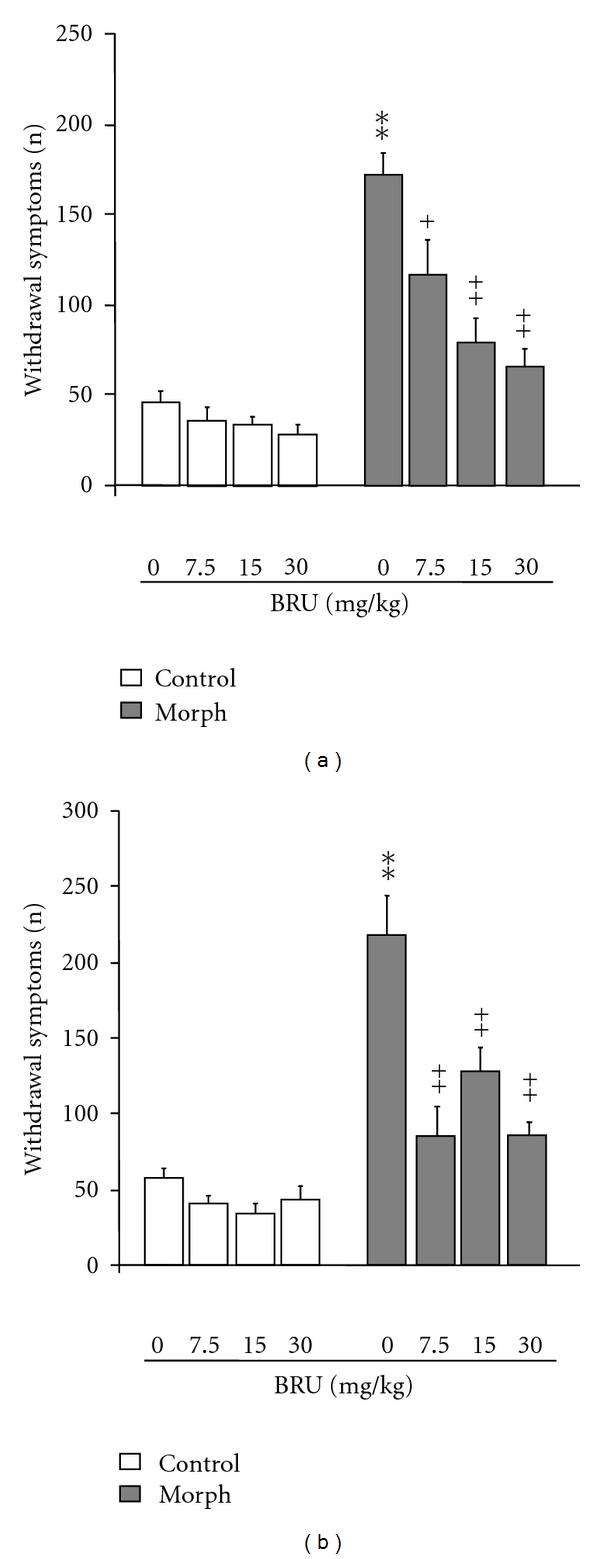
*Effects of different doses of Brugmansia arborea L. on the expression *(a)* and the acquisition *(b)* of morphine dependence.* Mice were treated twice daily for 5 days with saline or 10 mg/kg morphine treatment. On the sixth day, withdrawal syndromes were precipitated by injection of 5 mg/kg naloxone, 2 h after the last morphine injection. BRU (0, 7.5, 15, and 30 mg/kg) was administered 30 min before each morphine injection (acquisition) or prior to naloxone injection (expression). The withdrawal symptoms are given as a summary of the frequency of these somatic signs: jumping, rearing, forepaw tremors, and teeth chatter. Significant differences: **P < 0.01, compared to control; ^+^ *P* < 0.05, ^++^ *P* < 0.01, compared to morphine group.

Figures 4(a) and 4(b) show the effects of BRU on the expression and the acquisition of morphine dependence, respectively. Repeated administration of morphine produced physical dependence as assessed by the summary of characteristic set of behavioural responses including jumping, rearing, forepaw tremor, and teeth chattering [*F*(1,18) = 29.933; *P* < 0.001], following naloxone challenge.

As shown in Figure 4(a), acute administration of BRU 30 min prior to naloxone injection significantly decreased the expression of morphine dependence, as assessed by the summary of the frequencies of the signs of withdrawal syndrome compared with frequencies of withdrawal manifestations of morphine-dependent mice treated with vehicle [*F*(7.74) = 10.518; *P* < 0.001].

Particularly, the *post hoc* analysis revealed a statistically significant and dose-dependent effect at all doses tested (7.5, 15, and 30 mg/kg) (*P* < 0.001) (Figure 4(a)). No difference was observed in control mice treated with all doses of BRU when compared to vehicle (*P* > 0.05).

Figure 4(b) shows the effects of repeated administration of BRU on the development of morphine dependence. Pretreatment of the mice with BRU, 30 min before each morphine injection, attenuated the development of the characteristic signs of withdrawal, reported as total signs [*F*(7,74) = 17.774; *P* < 0.001] (Figure 4(b)). Indeed, the *post hoc* analysis revealed a statistically significant effect both at the highest dose of 30 mg/kg (*P* < 0.01) and at the lowest BRU doses tested, of 7.5 and 15 mg/kg (*P* < 0.01). The mice treated only with BRU (7.5, 15 and 30 mg/kg) did not show significant difference in withdrawal symptoms compared to the control group (*P* > 0.05) (Figure 4(b)).

### 3.5. Effects of FA and FNA on the Expression and the Acquisition of Morphine Dependence

**Figure 5:**
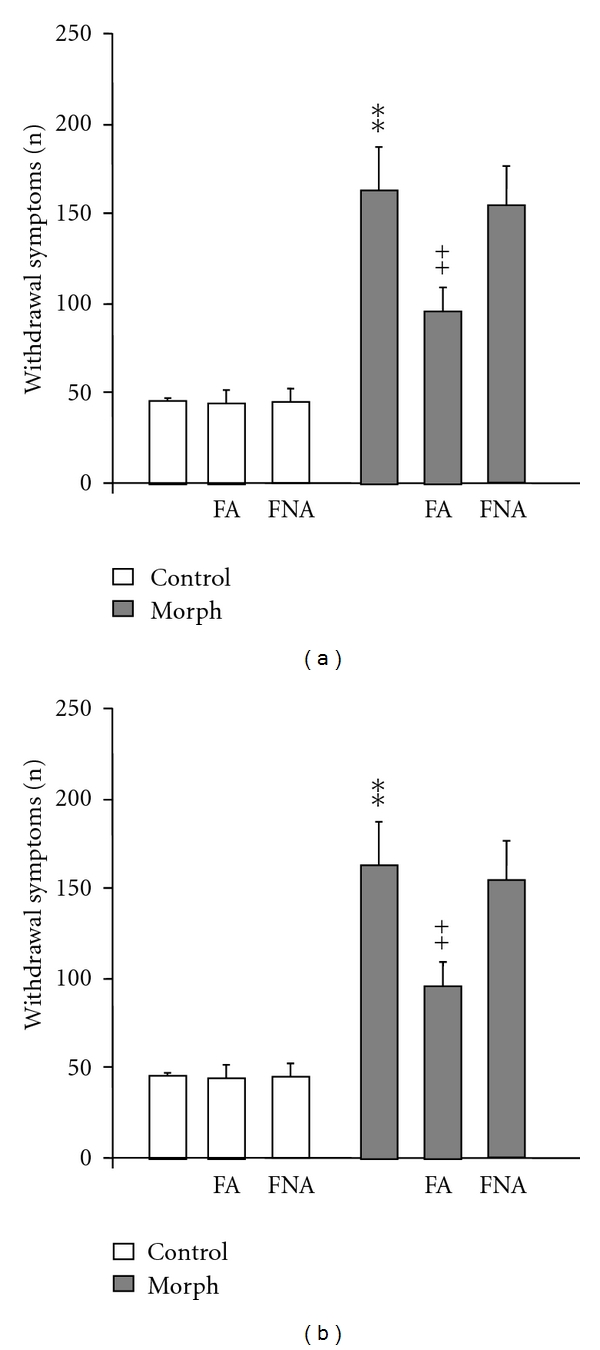
*Effects of FA and FNA on the expression *(a)* and the acquisition *(b)* of morphine dependence.* FA (5.5 mg/kg) and FNA (24.5 mg/kg) were administered 30 min before each morphine injection twice daily for 5 days (acquisition) or prior to naloxone injection (expression). On the sixth day, withdrawal syndromes were precipitated by injection of 5 mg/kg naloxone, 2 h after the last morphine injection. The withdrawal symptoms are given as a summary of the frequency of these somatic signs: jumping, rearing, forepaw tremors, and teeth chatter. Significant differences: **P < 0.01, compared to control; ^++^ *P* < 0.01, compared to morphine group.

Overall analysis of variance revealed a statistically significant treatment effect [expression: *F*(5,54) = 12.265, *P* < 0.001; acquisition: *F*(5,54) = 11.418, *P* < 0.001].  *Post hoc* analysis revealed that both single-dose administration of FA (5.5 mg/kg), 30 min before naloxone injection on the test day, and its repeated injection 30 min before each morphine treatment, produced significant decrease in the expression and the acquisition of morphine dependence (*P* < 0.01), respectively, as compared to vehicle group in morphine-treated mice (Figures 5(a) and 5(b)). In fact both acutely and repeatedly FA was able to suppress significantly the naloxone-precipitated withdrawal symptoms by about 50% in the morphine-dependent mice (*P* < 0.001). Conversely single or repeated administrations of FNA (24.5 mg/kg) were ineffective to prevent or treat morphine dependence in mice (*P* > 0.05) (Figures 5(a) and 5(b)). On the other hand, treatment with FA or FNA in nondependent mice did not affect naloxone-precipitated withdrawal symptoms elicited by single or repeated injection as compared to the control group (*P* > 0.05) (Figure 5).

### 3.6. Effects of Single Alkaloids on the Expression and the Acquisition of Morphine Dependence

**Figure 6:**
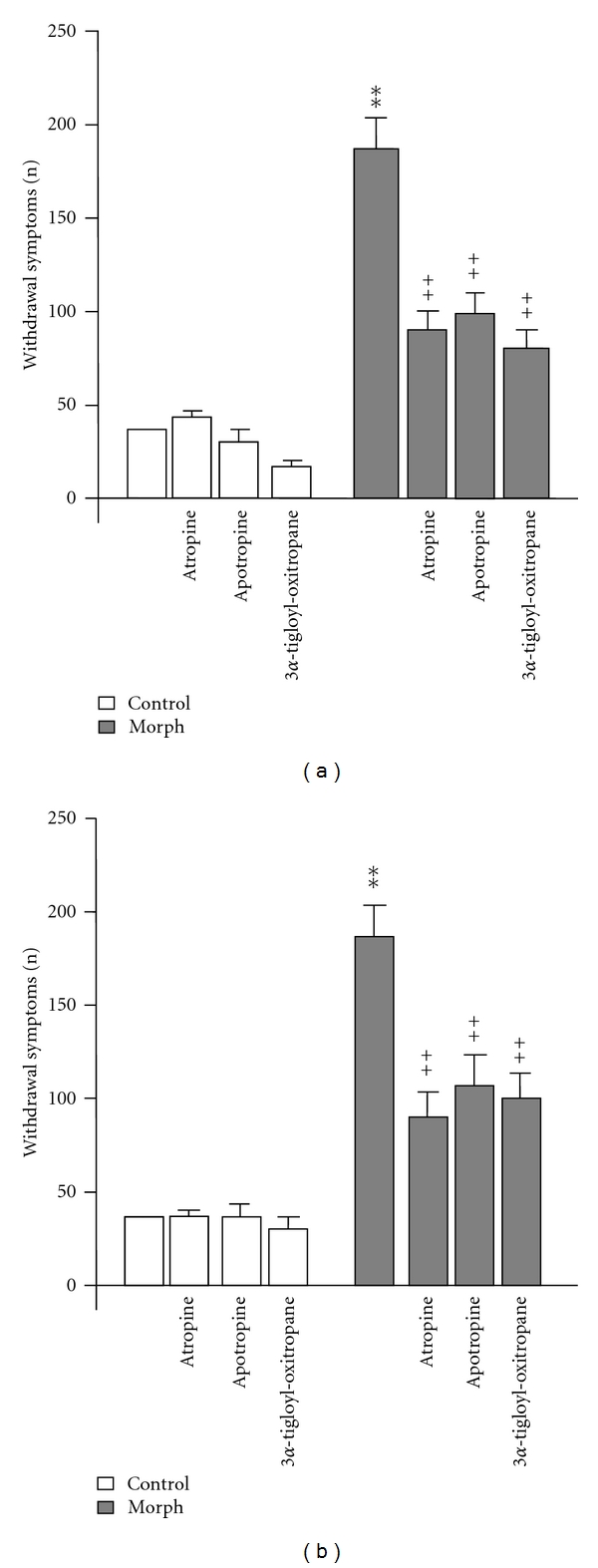
*Effects of single alkaloids on the expression *(a)* and the acquisition *(b)* of morphine dependence.* Atropine, apoatropine, and 3*α*-tigloyl-oxitropane were administered i.p. at the dose of 2.2, 1.8, and 1.5 mg/kg, respectively, 30 min before each morphine injection (acquisition) or prior to naloxone injection (expression) (*n* = 10 animals for each group). The withdrawal symptoms are given as a summary of the frequency of these somatic signs: jumping, rearing, forepaw tremors, and teeth chatter. Significant differences: **P < 0.01, compared to control; ^++^ *P* < 0.01, compared to morphine group.

As shown in Figure 6(a), atropine, apoatropine, and 3*α*-tigloyl-oxitropane (2.2, 1.8, and 1.5 mg/kg resp., i.p.) administered 30 min prior to naloxone injection inhibited about 50% naloxone-precipitated withdrawal symptoms compared with those of morphine control group [*F*(7,56) = 31.552, *P* < 0.001]. Overall analysis of variance revealed that repeated coadministration of atropine, apoatropine, and 3*α*-tigloyl-oxitropane with morphine decreased significantly the frequencies of the signs of withdrawal syndrome compared with frequencies of withdrawal manifestations of morphine-dependent mice treated with vehicle [*F*(7,56) = 25.192, *P* < 0.001]. Indeed, the *post hoc* analysis revealed a statistically significant effect for all alkaloids tested (*P* < 0.01) (Figure 6(b)).

The mice acutely or repeatedly treated only with atropine, apoatropine, and 3*α*-tigloyl-oxitropane did not show significant difference in withdrawal symptoms compared to control animals (*P* > 0.05) (Figures 6(a) and 6(b)).

## 4. Discussion

The present study has revealed the presence of three tropane alkaloids in the methanol extract of *Brugmansia arborea*. These compounds are usual in the genus [13], and no significant chemical differences have been found with the same species growing in America. Moreover it valuated the effects of BRU, a methanol extract of *B. arborea*, its alkaloidal and nonalkaloidal chromatographic fractions, or isolated alkaloids on both the acquisition and the expression of morphine tolerance and physical dependence in mice. The data demonstrate that administration of BRU at high dose attenuates the expression of morphine tolerance by increasing the antinociceptive response, but does not attenuate its acquisition. On the other hand, neither alkaloidal and nonalkaloidal fractions, nor pure alkaloids have been proven effective in preventing or countering the tolerance to the analgesic effect of morphine.

Conversely, administration of BRU attenuates both the expression and the development of morphine dependence by reducing the naloxone-induced behavioural and vegetative withdrawal symptoms in a dose-dependent manner. Indeed, BRU was effective in reducing the incidence of withdrawal symptoms in morphine-dependent mice. These data assume greater importance when it is considered that, historically, withdrawal symptoms were believed to have a major role in the relapse to drug-taking behaviour after drug abstinence [14]. BRU effects appear to be due to its alkaloidal fraction, as demonstrated by the effectiveness of single alkaloids in preventing and countering the onset of withdrawal symptoms induced by naloxone injection. On the other hand, nonalkaloidal fraction was ineffective in reducing the incidence of withdrawal symptoms in morphine-dependent mice.

The results of this study support and extend previous findings that central cholinergic receptors participate in the expression and acquisition of opiate withdrawal symptoms [15].

On the other hand, central cholinergic neurons have long been suggested to mediate many of the signs and symptoms of opiate withdrawal [15].

In fact pharmacological blockade of central muscarinic receptors by antagonists, such as scopolamine, has been widely used in treatment of drug abuse, especially in opioid addiction [16, 17]. Moreover it was also demonstrated the role of cholinergic system on morphine reward properties. Indeed blockade of muscarinic receptors by different doses of atropine into basolateral amygdala abolished morphine-induced place preference in rats [18].

The molecular and neurobiological mechanisms underlying the attenuation of morphine dependence by alkaloids present in *B. arborea *could be related to their direct blockade of muscarinic cholinergic receptors, which have been shown to interact with opioid receptor signalling [19].

Another possibility is that *B. arborea *attenuation of morphine dependence is due to its indirect effects on the mesocorticolimbic dopaminergic pathway. The decreased dopaminergic activity in the VTA induced by morphine withdrawal increases the activity of noradrenalinergic and cholinergic systems, which are mainly involved in morphine withdrawal symptoms.

Recent researches suggest a possible role for M5 receptors for the treatment of opiate addiction, based on M5 AChR brain region localization and involvement in the regulation of striatal dopamine release and in rewarding brain stimulation [20].

Moreover it was demonstrated that VTA ACh levels played a causal role on drug seeking and reward, and these effects were strongly attenuated by local infusion of a muscarinic antagonist, like atropine [21, 22]. Therefore *B. arborea* alkaloids, based on their tropanic structure, might prevent and counter morphine dependence by the reduction of ACh levels on VTA brain area.

Finally, DA system plays a critical role in drug craving and relapse, conditions that occur with dependence and withdrawal. Administration of D1 and D2 receptors agonists attenuated somatic withdrawal signs [23–25]. Therefore *B. arborea *extract might attenuate morphine dependence by acting directly on the mesocorticolimbic dopaminergic pathway, since it has been demonstrated the affinity of the extract for D1 and D2 receptors [5].

In conclusion, the positive effects of *B. arborea* extract and its pure alkaloids in the expression and development of morphine dependence encourage the use of the plant for the treatment of opioid addiction.
